# Short-term in vivo morphological changes of amniotic membrane after fibrin glue-assisted pterygium surgery on anterior segment optical coherence tomography: a case presentation

**DOI:** 10.1186/s12886-017-0576-2

**Published:** 2017-10-03

**Authors:** Jun Hyuk Son, Su-Ho Lim

**Affiliations:** 1 Department of Ophthalmology, Yeungnam University College of Medicine, Daegu, Republic of Korea; 2 Department of Ophthalmology, Daegu Veterans Health Service Medical Center, #60 Wolgok-Ro, Dalseo-Gu, 704-802 Daegu, Republic of Korea

**Keywords:** Amniotic membrane, Anterior segment optical coherence tomography, Case report, Oct, Pterygium

## Abstract

**Background:**

The evaluations of morphological changes of amniotic membrane (AM), even after successful AM transplantation surgery without complications, may be difficult. Moreover, there was no report regarding morphological changes after fibrin glue-assisted AM transplantation with pterygium excision. Here, we highlight and describe the use of spectral domain optical coherence tomography (OCT) for the evaluation of the morphological features of amniotic membrane (AM) and of associated in vivo structural changes after fibrin glue-assisted pterygium surgery.

**Case presentation:**

All three patients underwent cryo-preserved AM transplantation using the permanent inlay technique (epithelial side up) with fibrin glue. In vivo morphological changes of AMs were evaluated using a spectral domain OCT equipped with an anterior segment imaging module (RTVue-100, Optovue, Inc., Fremont, CA, USA). Anterior segment OCT examinations demonstrated morphological changes, that is, re-absorption of fibrin glue or subconjunctival hemorrhage, migration of epithelium, and integration of AM into sclera, of AMs over first postoperative months.

**Conclusions:**

Anterior segment OCT might provide additional structural information, including quantitative and qualitative data, on AMs after pterygium surgery as compared with conventional slit-lamp examination.

## Background

The amniotic membrane (AM), the avascular innermost layer of the placenta, promotes wound healing, reduces pain, and minimizes inflammation. Thus, AM is widely used to treat various ophthalmic conditions, especially those associated with ocular surface reconstruction [1]. AM thicknesses depend on methods of manufacture (cryopreserved vs. dry AMs) and the majority of dyes (indocyanine green, rose bengal, and trypan blue) strongly stain AMs for 24 h after application [2]. Subsequently, clinical evaluations of morphological changes of AMs, even after successful AM transplantation surgery without complications, may be difficult by these membrane properties.

Anterior segment optical coherence tomography (AS-OCT) is a noncontact technology that can allow detailed structural analysis at high resolution and reproducibility in various ocular surface diseases [3]. In these contexts, Nubile et al. demonstrated multiple layers of amniotic membrane could integrate into corneal stroma and resultantly increase corneal thickness by confocal microscopy and AS-OCT [4]. However, there was no report regarding morphological changes after fibrin glue-assisted AM transplantation with pterygium excision. Here, we describe for the first time in vivo morphological changes of AMs after fibrin glue-assisted AM transplantation with pterygium excision.

## Case presentation

All participants gave their informed consent to participate in this study, which was conducted in accordance with the tenets of the Declaration of Helsinki.

### Case 1

**Fig. 1:**
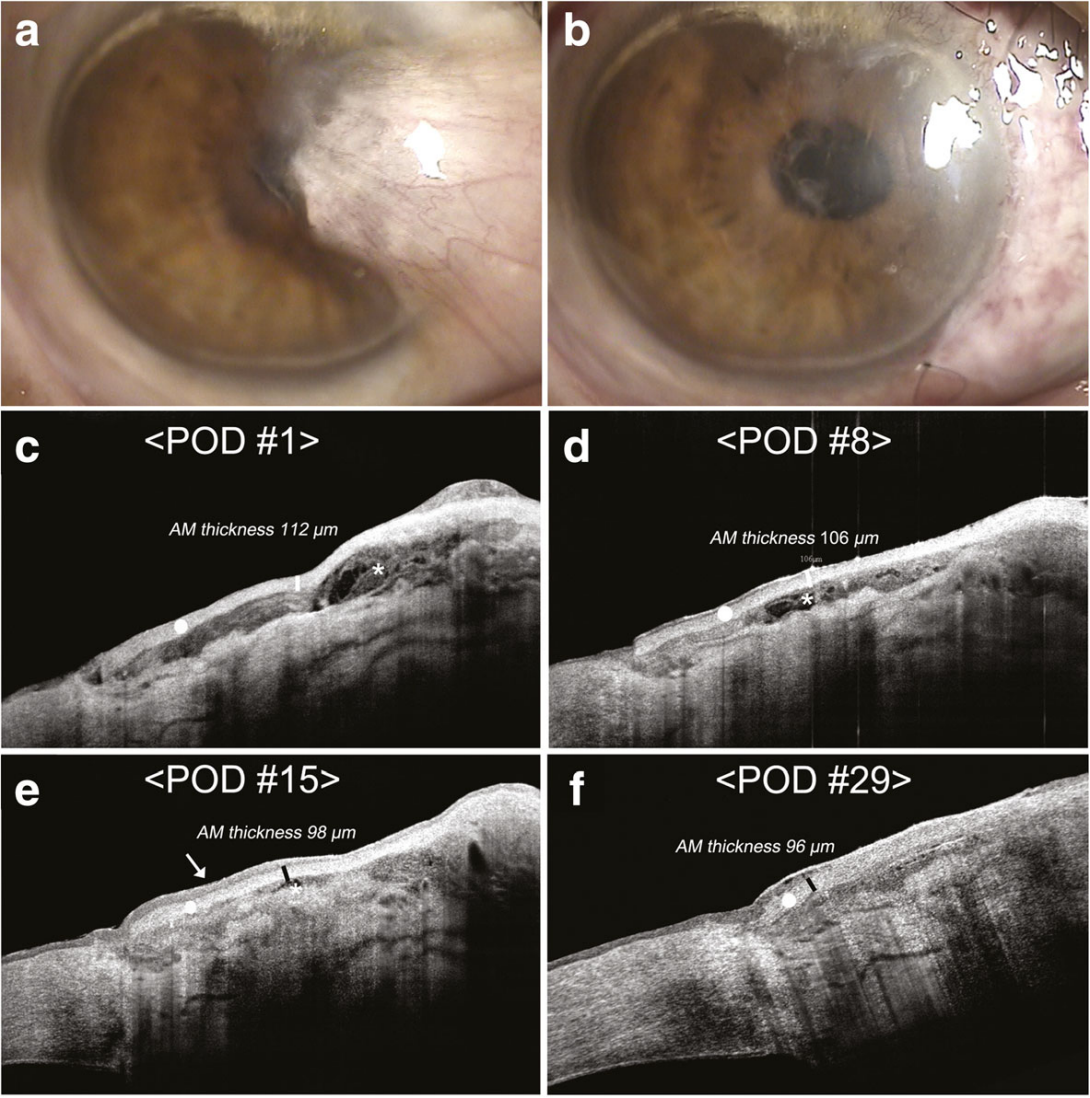
Clinical findings in Case 1. Anterior segment photograph of Case 1 before surgery (**a**) and the immediate postoperative image (**b**). Morphological changes of amniotic membrane (AM) after fibrin glue-assisted pterygium surgery in Case 1 (**c**-**f**). Anterior segment optical coherence tomography (AS-OCT) revealed thick fibrin sealant materials (asterisk) under grafted AM (white circle) on postoperative day 1 (**c**), which gradually diminished over 2 weeks after surgery (**d**, **e**). Complete re-epithelialization over the grafted AM was observed at 15 days postoperatively (arrow). Integrated amnion within sclera underwent progressive changes (decreased hyper-reflectivity of AM, decreased dead space under AM, and even (uniform) distribution of epithelium over AM) at 1 month postoperatively (**f**)

A 65-year-old Korean male patient (Case 1) visited the authors’ clinic with conjunctival injection in his right eye. He had undergone scleral buckling surgery due to rhegmatogenous retinal detachment 7 years earlier at another hospital. He reported taking metformin for diabetes. His uncorrected visual acuity was counting fingers in the right eye and 20/20 in the left, right eye was improved to 20/200 with pinhole. A slit-lamp biomicroscopic examination showed a thick fibrovascular ingrowth in the cornea, which extended across the pupil and obscured vision in his right eye (Fig. 1a) Thus, we decided to perform pterygium excision with a fibrin glue-assisted AM transplantation. In the operation room, the cryo-preserved AM was grafted using a permanent, inlay technique (epithelial side up) with fibrin glue (Greenplast Kit®, Green Cross Inc., Seoul, Korea) by mixed use of component. The AM was secured to host tissue using two additional anchoring sutures (10–0 nylon) parallel to limbus (Fig. 1b). Fourier-domain AS-OCT (RTVue-100, Optovue, Inc., Fremont, CA, USA) using 1310-nm examination revealed thick fibrin glue materials under the grafted AM on postoperative day 1 (POD1) and a gradual reduction over the first 2 postoperative weeks. Measured AM graft thickness were 112 μm on POD1, 106 *μm* on POD 8, and complete re-epithelialization over the grafted AM was observed at POD15. Integrated amnion within sclera underwent progressive changes over the first postoperative month (Fig. 1c-f). At 1 month postoperatively, uncorrected visual acuity improved to 20/50 and this improved to 20/40 with pinhole. Intraocular pressures (IOP) were measured using a Goldmann tonometer to be 14 mmHg in the right eye and 17 mmHg in the left eye at 1 month postoperatively. And we did not observe difference in the amniotic membrane integrated into the sclera of the patient that had undergone the scleral buckling compared to Case 2 and Case 3.

### Case 2

**Fig. 2:**
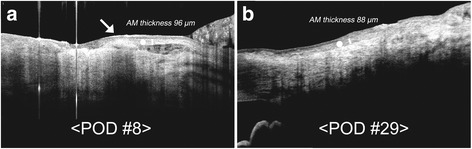
Anterior segment optical coherence tomography images from Case 2. AS-OCT showed corneal epithelium migrated over the grafted amniotic membrane (AM) at 1 week postoperatively (**a**, arrow), stroma of the AM (white circle) had integrated into sclera at 1 month postoperatively after excision of recurrent pterygium in this 74-year-old male patient (**b**)

A 74-year-old Korean male patient visited our clinic complaining of ocular discomfort in his right eye. He had no significant medical history, was not on any medication, and his ophthalmic history was unremarkable with no history of prior ocular surgery or any significant ocular disease. Slit-lamp biomicroscopic examination showed mild posterior blepharitis and primary pterygium in the right eye. Uncorrected visual acuity was 20/20 in both eyes, and IOP was 14/15 mmHg. He was also treated by pterygium excision and amniotic membrane transplantation using fibrin-glue (without suturing). On POD7, AS-OCT showed corneal epithelium had migrated over the grafted AM, and at 1 month postoperatively AM stroma had integrated into sclera (Fig. 2).

### Case 3

An 82-year-old Korean male patient presented at our clinic for ocular injection. The patient’s ocular history included cataract surgery (phacoemulsification) in both eyes and ocular surface disease treated with cyclosporine ophthalmic emulsion 0.05% twice daily. His medical history was significant for stroke more than 10 years previously. His current medications included aspirin and clopidogrel. His best-corrected visual acuity was 20/20 in the both eyes, and his IOP was 17/19 mmHg. Slit-lamp biomicroscopic examination revealed pterygium in his right eye but no other ocular comorbidity.

**Fig. 3:**
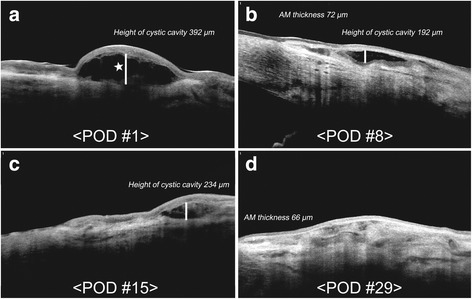
Anterior segment optical coherence tomography images from Case 3. **a**-**d** show re-absorption of subconjunctival hemorrhage and fibrin glue material under the amniotic membrane (AM) over the 4 weeks after fibrin glue-assisted pterygium surgery in this 82-year-old male patient. Bullous detachment of the AM with fluid collection below (star) was noticed on the day after surgery (**a**), but these decreased gradually and epithelial migration was complete at 4 weeks under close follow-up by AS-OCT (**b**-**d**)

In this patient, massive subconjunctival hemorrhage was encountered on the day following uneventful pterygium surgery with fibrin glue assisted permanent AM transplantation. At 4 weeks postoperatively, AS-OCT images revealed re-absorption of the subconjunctival hemorrhage and fibrin glue material under the AM. Bullous detachment of the AM and fluid collection under the membrane were noticed at POD1, but under close follow-up, these were found to decrease gradually, and at 4 weeks AS-OCT showed epithelial migration was complete (Fig. 3).

## Discussion

AMs has been used to manage many ocular surface diseases. However, slit-lamp examination only provides subjective information of morphological changes under grafted AMs and lacks accuracy [5], Another study also reported that slit-lamp beam measurements of pterygium can be difficult to reproduce accurately [6].

Fourier domain AS-OCT provides a depth resolution of 5 μm with a speed of 26,000 axial scans per second. Furthermore, AS-OCT provides accurate clinical assessment of extension of pterygia onto the cornea, and may be useful for research purposes [6]. These recent developed tools provide high-resolution images of pterygium and pinguecula, and clearly depict anatomical relationships between corneal tissues and lesions [7]. In these contexts, Soliman and Mohamed reported that AS-OCT might help to decrease recurrence rates after pterygium excision by providing more accurate evaluations of pathologic lesions [7]. However, to date there was no report regarding morphological changes after fibrin glue-assisted AM transplantation with pterygium excision.

To the best of our knowledge, this is first report on AM morphological changes after pterygium surgery with fibrin glue-assisted AM transplantation as determined by AS-OCT. In our cases, AS-OCT provided detailed structural analysis and depicted morphological AM changes after surgery. As shown in cases, AS-OCT provided AM thickness profiles after surgery. Ramos et al. [3] also concluded that AS-OCT accurately quantifies diseased corneal thickness, and similarly, Kheirkhah et al. [8] reported changes of conjunctival graft thickness after pterygium surgery using AS-OCT findings are compatible to those of the present study.

In our three patients, complete re-epithelialization were observed by AS-OCT within 1–4 weeks after surgery. In a previous confocal microscopic study, regenerating epithelium was observed to migrate over underlying AMs and this process was complete after a mean 15 days (range: 1–4 weeks) for corneal ulcers treated by multilayer AM transplantation [4], which concurs with our findings. Thus we suggest AS-OCT provides additional information about grafted AMs, especially with respect to morphological changes.

There are a few limitations in this study. First, we could not conclude the relationship between the volume of blood and fibrin glue below the amniotic membrane, and the time taken for the amniotic membrane to integrate into the sclera. Second, this study had small numbers of patients (*n* = 3) and short-term follow-up period. And the authors could not conclude the time of total integration or dissolving of the AM. One histopathologic study reported the AM integration in 11 of 14 patients up to 77 weeks after AMT [9]. Thus, further long-term and large observational studies are needed to clarify the timing of total integration/or dissolving of the AM.

Although, conventional slit-lamp biomicroscopic examinations allow direct viewing of the whole cornea and conjunctiva, comparisons performed during follow-up period are rather subjective [5, 10]. Moreover, compared to the simpler and cheaper method of fluorescein staining to check epithelial healing; AS-OCT does not seem to be a practical tool in monitoring regular, uncomplicated pterygium surgeries. Anterior segment photography may also used but these two modalities cannot provide detail of the anatomy below grafted AMs. However, on the other hand, AS-OCT images are objective, highly reproducible and can provide detailed anatomical information below AMs, and thus, we believe AS-OCT may be more useful for evaluations performed after pterygium surgery in a large clinical setting where multiple doctors might follow a single patient [10].

## Conclusion

In summary, as compared with conventional slit-lamp examination, AS-OCT provides additional structural information on AMs after surgery. The AS-OCT examinations performed on our three patients demonstrated AM morphological changes during the first months after surgery, that is, the re-absorption of fibrin glue and subconjunctival hemorrhage, migration of epithelium, and integration of AM into sclera. We hope that these case reports help physicians better understand AM changes after surgery.

## Abbreviations

AM: Amniotic membrane
AS-OCT: Anterior segment optical coherence tomography
IOP: Intraocular pressure
POD: Postoperative day
